# Impact of Wheat on Soybean Cyst Nematode Population Density in Double-Cropping Soybean Production

**DOI:** 10.3389/fpls.2021.640714

**Published:** 2021-05-10

**Authors:** Leonardo F. Rocha, Mirian F. Pimentel, John Bailey, Terry Wyciskalla, Dan Davidson, Ahmad M. Fakhoury, Jason P. Bond

**Affiliations:** ^1^School of Agricultural Sciences, Southern Illinois University, Carbondale, IL, United States; ^2^JCB Ag Research, Effingham, IL, United States; ^3^Wyciskalla Consulting, Nashville, IL, United States; ^4^Davidson Consulting, Stanton, NE, United States

**Keywords:** SCN, *Heterodera glycines*, soybean diseases, crop rotation, plant-parasitic nematodes, integrated pest management, nematode suppression, suppressive soils

## Abstract

Double-cropping is defined as producing more than one crop on the same parcel of land in a single growing season. It is reported to have many benefits when incorporated in cropping systems, including improving soil health. In some double-cropping systems, soybean is planted following winter wheat. The soybean cyst nematode (SCN) (*Heterodera glycines* Ichinohe) is a major soybean pathogen, and several reports suggest suppressive effects of wheat on SCN populations. Field trials were conducted from 2017 to 2018 to investigate the effect of wheat on SCN populations in double-cropping soybean. Nine fields with three levels of initial SCN populations (low, moderate, and high) were selected in Illinois. Wheat was planted in strips alternating with strips-maintained weed-free and under fallow over winter and early spring. Soybean was planted in all strips after wheat harvest. SCN egg densities were acquired at four time points: wheat establishment, post-wheat/pre-soybean, mid-soybean (R1 growth stage or beginning of flowering), and post-soybean harvest. Wheat strips reduced SCN egg densities compared with fallow strips at the R1 stage (−31.8%) and after soybean harvest (−32.7%). Double-cropping soybean with wheat has the potential to suppress SCN field populations and is a system with the potential to provide additional farm income. This study is meant to be a first step toward a better understanding of the mechanisms that govern the suppression of SCN by wheat.

## Introduction

Many modern cropping systems are based on the cultivation of single crops in yearly rotations. However, fall-planted crops have been increasingly incorporated due to resulting weed suppression and enhanced environmental stewardship (Leslie et al., 2017). Double-cropping is defined as producing more than one crop on the same parcel of land in a single growing season. This system is being adopted as one of the multiple strategies used to increase biomass productivity in agricultural lands and to help supply the increasing demand for food and feed (Caviglia and Andrade, 2010). Compared with monocropping, double-cropping systems stand out in capturing radiation and rainfall and more effectively using inputs, thus allowing to optimize the exploitation of the potential productivity of fields (Caviglia et al., 2004; Heggenstaller et al., 2009). In the United States, crops established in the fall are harvested in late spring and followed directly by a second warm-season crop (Heggenstaller et al., 2009). Due to the shorter growing season and fewer winter crop options, this practice is less adopted in the Midwest compared with southern and mid-southern areas of the United States (Heggenstaller et al., 2009).

Double-cropped soybeans are commonly planted in fields following the harvest of winter wheat in mid to late June (Nafziger, 2009). Although two distinct crops are grown in one season, farmers are still recommended to rotate summer crops, as double-cropping is not considered a crop rotation. In Illinois, double-cropping has been more successful in the southern portion of the state, where favorable weather conditions allow earlier wheat harvest and soybean planting, while warmer weather in the fall season allows winter crops to grow for an extended window before being exposed to freezing temperatures (Nafziger, 2009). Shorter soybean growing windows due to late wheat harvest may lead to reduced vegetative development before flowering and consequential yield reduction (Nafziger, 2009). Therefore, the main factors reducing yield in double-cropping soybean are later planting dates, water deficit, reduced radiation/photoperiod, the effect of winter crop residues, nutrient deficits, and susceptibility to early frost (Hansel et al., 2019). Nevertheless, the benefits of double-cropping include improvement of soil chemical and physical properties, control of erosion, reduction in tillage requirements, economic value of two crops in the same season, and improved soil microbial and faunal activity (Peralta et al., 2018).

The soybean cyst nematode (SCN) (*Heterodera glycines* Ichinohe) is a major plant-parasitic nematode on soybean, and it is widely distributed in all major soybean production areas of the United States (Niblack and Tylka, 2008). In Illinois, SCN is present in more than 80% of the fields (Niblack et al., 2009) and in every county (Tylka and Marett, 2017). SCN causes losses of up to 60% in susceptible cultivars (Hershman, 2014), and often losses of up to 30% occur without showing noticeable aboveground symptoms (Mueller et al., 2016; Tylka and Marett, 2017). In a survey from 2010 to 2014, soybean yield losses caused by SCN nationwide were estimated to be twice those caused by other diseases combined (Allen et al., 2017). In order to reduce losses, several management practices are recommended, including using resistant and tolerant cultivars, crop rotation with non-hosts, weed management, seed-applied nematicides, and biological control products (Niblack and Tylka, 2008; Niblack et al., 2009; Wight et al., 2011; Mueller et al., 2016). Currently, most commercial cultivars available within Illinois share a common source of resistance, the plant introduction (PI) 88788. Over the years, the lack of rotation among sources of resistance led to the selection of SCN populations able to reproduce on available resistant soybean cultivars (Niblack and Tylka, 2008). This has resulted in the reduction of management options available for farmers.

Several research reports show significant suppression effects of wheat on SCN populations, but many do not separate effects of tillage from cover crops or assess the effect of the initial SCN population on the system (Baird and Bernard, 1984; Koenning, 1991; Hershman and Bachi, 1995; Long and Todd, 2001; Warnke et al., 2006; Wight et al., 2011; Bernard, 2018). In addition, most wheat/SCN research was conducted in the midsouth and southern United States and in small plot trials. Farmers commonly use wheat in double-cropping soybean systems in Illinois, and this cropping system has the potential to be expanded to soybean farming areas in higher latitudes of the United States (Zabel et al., 2014; Seifert and Lobell, 2015). Therefore, the objective of this work was to assess the effect of wheat on SCN population densities in double-cropping soybean in farming conditions in Illinois.

## Materials and Methods

### Field Establishment

**FIGURE 1:**
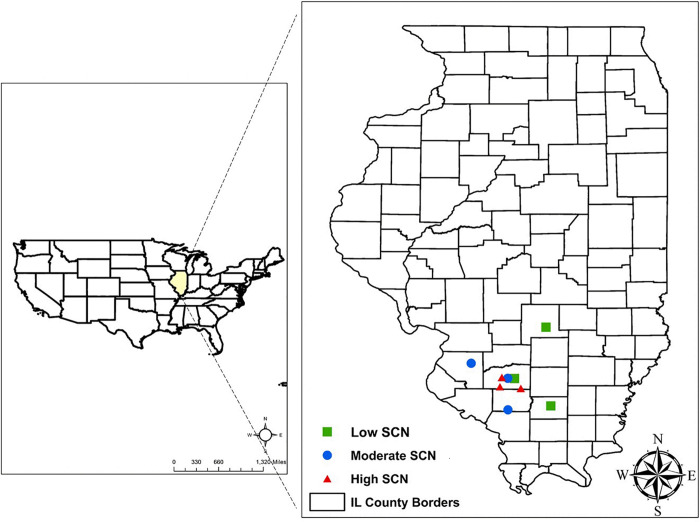
Field locations selected in Illinois to study the impact of wheat on soybean cyst nematode (SCN) in double-cropping soybean. SCN population densities were defined as high SCN when having >6,500 eggs/100 cm^3^ of soil, moderate for 2,000–6,500 eggs/100 cm^3^, and low when <2,000 eggs/100 cm^3^.

This study was conducted in row crop fields provided by farmers, located in commercial farms in Illinois. The fields were selected based on the results from an initial SCN field survey performed in 2017 (Supplementary Table 1). The SCN population densities were determined in 22 fields in seven counties. From this survey, nine fields were identified for use in this study, including three locations each for low, moderate, and high initial SCN population densities (Figure 1). Fields with >6,500 eggs/100 cm^3^ of soil were classified as high SCN. Those with 2,000–6,500 eggs/100 cm^3^ were classified as moderate, and fields with <2,000 eggs/100 cm^3^ were classified as low SCN (adapted from the University of Illinois Extension, 2014). The SCN HG (*H. glycines*) type was determined for field populations as described by Niblack et al. (2002). HG type testing is a greenhouse assay that quantifies how much SCN populations reproduce on sources of resistance available in soybean cultivars, replacing the race classification system. All field populations were HG type 2.5.7 with the exception of field 8, which was HG type 7 (Supplementary Table 1). HG type 2.5.7 has reproduction greater than or equal to 10% of that observed on the susceptible (Lee 74) on the soybean indicator lines PI88788, PI209332, and PI548316 (Cloud). HG type 7 indicates reproduction greater than or equal to 10% of the susceptible g only for PI548316 (Cloud) (Tylka, 2016). Location descriptions including initial SCN populations, rainfall, and soil chemical analysis are listed in Table 1.

**TABLE 1:** Field locations, initial SCN population densities, soil chemical properties, and cumulative rainfall.

Field	IL county	Soybean cyst nematode		Rainfall (mm)^4^	pH^1^	OM (%)	P^2^	K^2^	Mg^2^	Ca^2^	^3^CEC
		Egg density^5^	Level^6^				mg kg^–1^				
1	Perry	3,902	Moderate	680.72	6.3	2.7	27	85	65	1,134	7.6
2	St. Clair	2,140	Moderate	643.34	6.4	2.6	33	124	143	1,624	10.8
3	Washington	667	Low	790.45	5.7	2.7	16	85	77	1,161	9.1
4	Fayette	600	Low	710.69	5.3	2.1	33	76	68	826	8.1
5	Franklin	940	Low	670.56	6.8	2.8	32	101	195	1,350	9.4
6	Washington	7,700	High	790.45	7.0	2.1	50	85	90	1,526	8.6
7	Washington	8,858	High	790.45	6.1	2.0	57	91	71	994	7.0
8	Washington	8,626	High	790.45	6.5	2.3	32	116	86	1,239	8.4
9	Washington	3,909	Moderate	790.45	6.3	3.6	46	138	100	1,753	11.5

*^1^(1:1 H_2_O). ^2^Mehlich-3 (M3). ^3^Total cation exchange capacity meq/100 g. ^4^Average county rainfall during field trials (November 17 to October 18). ^5^Expressed in eggs/100 cm^3^. ^6^SCN population densities were defined as high SCN when having >6,500 eggs/100 cm^3^ of soil, moderate for 2,000–6,500 eggs/100 cm^3^, and low when <2,000 eggs/100 cm^3^.*

**FIGURE 2:**
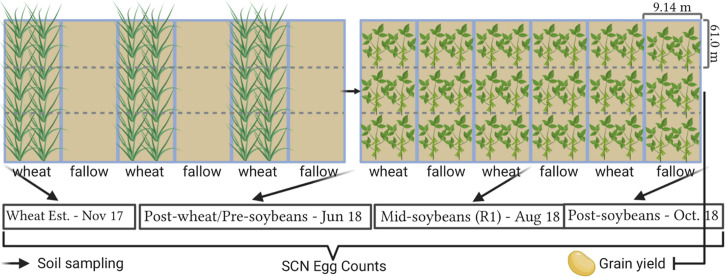
Field trial layout and sampling intervals. Plot representations are not in scale. Created with BioRender® (Biorender.com).

All fields were planted using commercial soybean cultivars with resistance to SCN (PI88788) in the previous summer (2017). In each location, the experimental design consisted of two treatments (WT: winter wheat and FL: fallow) with three replications. Each treatment was in strips (9.14 m wide × 182.9 m long), with three strips assigned for winter wheat and three strips that remained for fallow. Strips were subdivided into three subplots (9.14 m wide × 61.0 m long for soil sampling). Thus, the study consisted of a total of 18 subplots with a total of 1.1 hectares per location (Figure 2). Wheat was planted in fall 2017, and 2 weeks after emergence, wheat was terminated with herbicides to establish and maintain fallow strips. Herbicides were applied over winter to keep plots weed-free and prevent any potential SCN reproduction on volunteer soybeans and weeds, since more than a 100 weed species are reported to be potential hosts of SCN (Rocha et al., 2021). Soybean was planted in all subplots following wheat harvest in June 2018 using cultivars with resistance to SCN (PI88788), as this is a widely available resistance source in commercial cultivars, allowing thus to better simulate conditions experienced by soybean producers. Soybean was harvested in September 2018 and yield was assessed. All field operations from planting to harvest were conducted by producer collaborators across the entire study area at each location, with the exception of herbicide applications in fallow plots. A list of all soybean and wheat cultivars, planting dates, and fertilizers and herbicides used, as well as soil descriptions, is available in Supplementary Table 3.

### Soil Sampling and SCN Egg Density

The population densities of SCN were assessed at four intervals in this study. Soil samples were collected at wheat establishment (1: November 2017—2 weeks after wheat emergence or Feekes 2); in the window after wheat harvest and soybean planting (2: June 2018); at soybean growth stage R1, which denotes beginning of flowering (3: August 2018); and after soybean harvest (4: October 2018). From each location, a total of 18 soil samples were collected (2 treatments × 3 replicates × 3 subsamples per replicate) for each of the four sampling intervals. A total of 648 samples were collected and processed for this study.

Soil samples were collected from subplots at each of the four intervals using a cylindrical soil probe. Each sample was a composite of 20 soil cores (2.5 cm diam. × 20 cm deep) collected in a zigzag pattern, bulked in a bucket, sieved through a 6,350-μm pore mesh, and stored in a cooler for transport. Subsequent soil samples were collected approximately in the same area at each time point, as points were georeferenced, maintaining a 0.30-m foot distance from the previous sampling spot to avoid effects of disturbed soil on surrounding SCN egg densities. SCN cysts were extracted from 100 cm^3^ of soil by wet-sieving through nested 707- and 250-μm pore sieves. Eggs were extracted from the cysts and stained with acid fuchsin to facilitate visualization, and density was assessed using a counting slide (Chalex Corporation, Grasonville, MD, United States) under a Nikon SMZ-645 stereoscope. SCN population densities were expressed in eggs/100 cm^3^ of soil.

### Data Analysis

Statistical analyses were performed using SAS version 9.4 (SAS Institute Inc., Cary, NC, United States). Population densities of SCN were analyzed at each sampling interval using a generalized linear mixed model applying the GLIMMIX procedure with Poisson distribution and a log link function. Initial SCN population densities, winter rotation, and their interaction were considered fixed effects, whereas initial SCN population densities within field location, replications and treatments within subplots, and subplots within field location were considered random effects. When the main effects were significant (*P* < 0.05), the means were separated using Tukey’s HSD test (*P* = 0.05).

## Results

**FIGURE 3:**
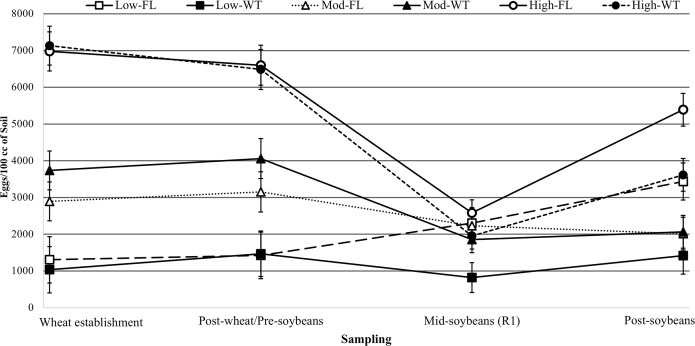
Egg densities (eggs/100 cm^3^ of soil) over time in soybean fields with low, high, or moderate initial SCN populations following winter wheat (WT) or fallow (FL). * Denotes significant differences in egg densities between winter options for all initial populations at that time point (*P* < 0.05).

Soybean cyst nematode population densities were enumerated at four sampling intervals to assess the effect of the winter option—wheat or fallow—on SCN egg densities and to monitor subsequent fluctuations in SCN population densities over time. The effects of initial SCN population densities (I), winter rotation (W), and the interaction between these two factors (I ^∗^ W) on egg densities are presented in Table 2. Fluctuations in field population densities over time are displayed in Figure 3.

**TABLE 2:** ANOVA table indicating the effect of SCN initial population density (I—low, moderate, or high^1^) and winter rotation (W—fallow or wheat) on the number of SCN eggs (eggs/100 cm^3^ of soil) at four time points throughout the soybean season.

SCN egg densities			
Source	*DF*	*F* ratio	*P* > *F*
**1: Wheat establishment**			
SCN initial pop. (I)	2	21.167	<0.0001*
Winter rotation (W)	1	0.749	0.3882
I * W	2	1.207	0.3020
**2: Post-wheat/pre-soybean**			
SCN initial pop. (I)	2	41.465	<0.0001*
Winter rotation (W)	1	0.540	0.4638
I * W	2	0.062	0.9399
**3: Mid-soybean (R1)**			
SCN initial pop. (I)	2	8.143	0.0004*
Winter rotation (W)	1	9.390	0.0026*
I * W	2	1.582	0.2093
**4: Post-soybean**			
SCN initial pop. (I)	2	16.387	<0.0001*
Winter rotation (W)	1	9.769	0.0021*
I * W	2	2.6416	0.0747
**Soybean yield**			
SCN initial pop. (I)	2	1.008	0.4288
Winter rotation (W)	1	59.898	<0.0001*
I * W	2	2.166	0.1192

*Indicates a significant effect of the factor on the tested variable (*P* < 0.05). ^1^SCN population densities were defined as high SCN when having >6,500 eggs/100 cm^3^ of soil, moderate for 2,000–6,500 eggs/100 cm^3^, and low when <2,000 eggs/100 cm^3^.

At wheat establishment, an initial SCN population density was determined to create a baseline for this study (Table 2). In the sampling interval following the wheat growing season and prior to soybean planting, the main factor driving SCN egg densities at wheat harvest was the field initial SCN population density (*F* = 41.465; *P* < 0.0001). Since wheat is a non-host of SCN, neither winter option (*F* = 0.540; *P* = 0.4638) nor factorial interaction (I ^∗^ W) (*F* = 0.062; *P* = 0.9399) affected SCN egg densities (Table 2).

At R1 soybeans (beginning of flowering), the initial SCN population density was a driver of SCN egg densities at mid-soybean (*F* = 8.142; *P* = 0.0004). The SCN egg density was also significantly affected by winter option (*F* = 9.390; *P* = 0.0026) (Table 2). In fact, SCN egg densities were reduced by 31.8% in wheat strips compared with fallow strips (Table 3). It is to be noted that the factorial interaction (I ^∗^ W) was non-significant (*F* = 1.582; *P* = 0.2093) on SCN egg densities (Table 2). At this interval, SCN population densities significantly dropped across fields and treatments, demonstrating an attrition of SCN populations throughout time (Figure 3).

**TABLE 3:** Effects of initial SCN population density (I—low, moderate, or high) and winter rotation (W—fallow or wheat) on SCN egg densities (eggs/100 cm^3^ of soil).

Factor	Level^1^	Wheat establishment			Post-wheat/Pre-soy			Mid-soybean (R1)			Post-soybean		
		Mean	Group	SE^2^	Mean	Group	SE	Mean	Group	SE	Mean	Group	SE
Initial pop.	Low	1,166.8	C	446.9	1,448.3	C	442.3	1,488.8	B	290.1	2,427.1	B	357.9
	Moderate	3,317.0	B	374.9	3,605.9	B	385.3	2,041.5	A	252.7	2,040.4	B	315.6
	High	7,056.3	A	374.9	6,543.0	A	385.3	2,268.5	A	252.7	4,504.0	A	315.6

Winter rotation	Fallow	3,726.2	A	326.9	3,944.3	A	332.4	2,347.3	A	218.0	3,628.8	A	269.7
	Wheat	3,969.5	A	326.9	4,207.2	A	329.2	1,601.1	B	215.9	2,442.3	B	269.7

Means followed by the same letter in the columns at each time of sampling are not different following Tukey’s HSD Test (*P* = 0.05). ^1^SCN population densities were defined as high SCN when having >6,500 eggs/100 cm^3^ of soil, moderate for 2,000–6,500 eggs/100 cm^3^, and low with <2,000 eggs/100 cm^3^. ^2^Standard error. Factorial interaction (I * W) was non-significant.

After soybean harvest, the results follow a trend similar to what was observed at the mid-soybean sampling interval (R1). Both initial SCN population density (*F* = 16.387; *P* < 0.0001) and winter option (*F* = 9.769; *P* = 0.0021) impacted SCN egg densities (Table 3). SCN egg densities in wheat strips were reduced by 32.7% compared with those in fallow strips. In addition, there was an overall upsurge in SCN population densities across fields and treatments, demonstrating an increase in SCN egg densities in later soybean reproductive stages. At this sampling interval, the factorial interaction (I ^∗^ W) was non-significant (*F* = 2.6416; *P* = 0.0747) on the SCN egg densities.

An overall increase in SCN egg densities was observed at harvest, but the SCN population recovery rate was slower in wheat strips in comparison with that in fallow strips (Figure 3). These results are confirmed when analyzing population ratios comparing end of soybean season egg counts to soybean planting (October 2018/June 2018). Such ratios allow to observe population dynamics throughout the soybean growing season. Fields with low initial SCN population experienced higher population increase rates (9.441) compared with fields with moderate (1.1183) and high (1.015) (*F* = 19.166; *P* < 0.001) initial SCN populations (Supplementary Table 2 and Figure 3). In fallow plots, the average population ratio was 4.999, whereas in double-cropping plots, those ratios were statistically lower and equal to 1.811 (*F* = 12.263; *P* = 0.0006) (Supplementary Table 2). These results support the conclusions drawn from the time point analysis, highlighting a reduction in SCN reproduction in double-cropping fields. A pattern of increase in SCN population density under fallow is especially observed in fields with high and low initial SCN population densities. In fields with a moderate population level, initial population densities were slightly higher in wheat plots at the beginning of the experiment, even though not statistically significant, and this was probably due to patchiness (non-homogeneity) in the distribution of SCN. In those fields, although final egg densities were similar, SCN population densities were reduced over time in wheat strips (Figure 3).

The winter option significantly impacted soybean yield (*F* = 59.89; *P* < 0.0001). Soybean yield was reduced by 300 kg per hectare (4.45 bushel/acre or 7.5%) on average in wheat strips compared with that in fallow strips (Table 4). There was no detected effect of the initial SCN population level (*F* = 1.01; *P* < 0.4288) nor of the factorial interaction (I ^∗^ W) on soybean yield (*F* = 2.17; *P* = 0.1192) (Table 2).

**TABLE 4:** Effect of initial SCN population level (low, moderate, or high) and winter option (winter wheat or fallow) on soybean yield (kg ha^–1^).

Factor (winter option)	Level^a^	Yield (kg ha^–1b,c^)	SE^d^
Initial SCN population	Low	3,763.3 A	336.5
	Moderate	3,384.0 A	336.5
	High	3,944.4 A	412.1
Winter option	Wheat	3,688.0 B	211.6
	Fallow	3,987.3 A	211.6

*^*a*^SCN population densities were defined as high SCN when having >6,500 eggs/100 cm^3^ of soil, moderate for 2,000–6,500 eggs/100 cm^3^, and low when <2,000 eggs/100 cm^3^. ^*b*^One bushel/acre = 67.25 kg ha^–1^. ^*c*^Means followed by the same letter in the columns for each factor do not differ by Tukey’s HSD test (*P* = 0.05). ^*d*^Standard error.*

## Discussion

Field trials were conducted with normal production practices in Illinois to assess the effect of wheat on SCN populations in double-cropping soybean. This study was conducted at locations further north than prior research. In addition, by expanding plot sizes and conducting trials in production fields, this study more closely simulates conditions experienced by producers. In soybean, SCN management is predominantly based on the use of resistant cultivars, crop rotation, and the application of nematicides *via* seed treatment. Management practices such as crop rotation and cover crops may reduce SCN populations, although SCN demonstrates the ability to survive in fields for years in the absence of growing soybean (Francl and Dropkin, 1986). This indicates that even a long-term rotation with non-hosts may not be sufficient to eliminate SCN from fields (Wight et al., 2011).

In this study, SCN egg densities were similar in wheat strips compared with fallow strips at wheat harvest. At that sampling interval, the main driver of SCN egg density was the initial SCN population densities. Furthermore, SCN egg densities did not fluctuate significantly during the wheat growing season, since wheat is a non-host of SCN, with comparable SCN population densities post-wheat harvest and at wheat establishment. Spring temperatures in Illinois were below average in 2018, especially in April, marking the second coldest record for this month for the state (Kerschner, 2018), possibly delaying SCN hatching and early development. Winter survival rates of SCN in the Midwest may attain 100%, which adds additional pressure on management practices aimed at maintaining or increasing soybean yield, since fields may be harboring high initial SCN inoculum at planting (Riggs et al., 2001). Data from double-cropping studies in the literature indicate that SCN population densities at soybean planting were not affected following wheat, yet plots with wheat residue were reported to show reduced SCN population densities at soybean harvest (Hershman and Bachi, 1995). Results from Rothrock and Kirkpatrick (2018) denoted no differences in SCN population densities in early season, late season, or postharvest among winter cover crops, including wheat. Others have shown potential suppressive effects of cover crops on SCN population densities at soybean planting. However, these studies allude to high initial SCN populations and exponential reproduction driving population densities at later stages, with yield losses being observed especially when susceptible cultivars are used (Long and Todd, 2001; McSorley, 2011).

At the R1 growth stage of soybean (beginning of flowering), our data reflected a reduction in SCN egg densities in wheat strips compared with fallow. At this sampling interval, the SCN egg density was affected by the initial SCN population densities. The literature suggests environmental factors, influence of wheat stubble and wheat root exudates, and mechanical interference with host recognition by SCN as possible factors leading to reduced SCN population densities where wheat preceded soybean. However, results from this field trial and from the literature demonstrate that the suppressive effects of wheat on SCN are not fully expressed at soybean planting, suggesting that wheat production alone may not explain all SCN suppression (Baird and Bernard, 1984; Jennings and Bernard, 1987; Hershman and Bachi, 1995). Wheat crop residues may as well lower soil temperatures at soybean planting and during the initial stages of development of the crop (Hansel et al., 2019). Even though SCN egg hatch, root penetration, and development can occur at a widespread temperature range, the rate of SCN growth, development, and reproduction is strongly influenced by temperature (Alston and Schmitt, 1988; Bernard, 2018). Although planting dates were similar in the current study, early maturity cultivars are often used in double-cropping fields, which could limit SCN reproduction due to reduced period with a host available.

Furthermore, egg density data at R1 growth stage of soybean indicate an attrition in SCN populations, since both fallow and wheat strips exhibited reduction in egg density at the R1 stage compared with the time of planting. Nematode attrition is commonly observed in field trials (McSorley and Dickson, 1989; Chen et al., 2006; Walthall et al., 2013), and it is characterized by a decrease in field population densities over time. Attrition is especially reported high during summer and is attributed to multiple biotic and abiotic factors. Fluctuations in populations of phytoparasitic nematodes are often driven by the availability and growth stage of susceptible hosts, environmental conditions, parasitism, and the production of harmful metabolites by competitors (Acedo et al., 1984).

Similar trends were observed at the soybean harvest sampling interval compared with those at R1 growth stage of soybean; SCN egg densities were reduced in wheat strips than in fallow strips. Likewise, in previous studies, fall-planted Italian ryegrass, rye, and oat led to reduced SCN populations at soybean harvest in the following year and were reported to have potential in helping farmers practicing continuous soybean production (Ackley, 2013). In this study, SCN field populations at soybean harvest interval increased compared with those at soybean R1 growth stage. Research indicates a peak in egg hatching and second-stage juvenile (J2) infectivity rates near August and with the expansion of the root system during the last vegetative growth stages. This can result in an exponential growth in field SCN populations (Yen et al., 1995). It is to be noted that, in this study, the population recovery rate was slower in wheat strips compared with that noted in fallow strips, resulting in reduced egg densities in double-cropping fields at harvest.

The diversification of cropping systems increases the richness of soil microbial communities and results in shifts in the core groups of fungi and bacteria (Peralta et al., 2018). Moreover, SCN can be parasitized by different nematophagous microorganisms, and research suggests an effect of changes in soil microbial communities on suppressing nematode populations in production fields (Tyler et al., 1987; Nour et al., 2003; Song et al., 2016). In a study conducted in Jackson, Tennessee, Bernard et al. (1997) reported a threefold increase in SCN female parasitism by fungi in September compared with that in July and August. Interestingly, in our study, this coincides with a similar time window where slower recovery rates of SCN populations were detected following wheat strips, suggesting that microbial antagonists might contribute to the suppressive effects of wheat. In similar studies, the effect of wheat suppressing SCN became more pronounced after multiple seasons. In fact, studies report that several years is needed before the impact of crop rotation or the use of cover crops was no longer impacting soil nematode communities. Long and Todd (2001) reported reductions in an SCN population 3–4 years after the establishment of a double-cropping system. Similarly, Tyler et al. (1987) reported that long-term tillage reduced SCN populations in the first 3 or 4 years after the establishment of their cropping system.

Soybean yield was reduced in fields with wheat as winter option. Results from the literature report both reduced and unaffected soybean yield following winter wheat, but overall, full season tends to yield more than double-cropping soybean, especially in high-yielding environments, where the prospect of maximized soybean yield decreases with increased wheat yield and delayed soybean planting date (Nelson et al., 2010; Hansel et al., 2019). Double-cropped soybean generally has higher total return compared to the full-season crop, but many producers also adopt this system to increase cash flow and as a risk diversification strategy, reducing the dependency on a single commodity market (Holshouser, 2015). While maximized wheat production may lead to reduced soybean yield, high-yielding wheat is a prerequisite for a profitable double-cropping system (Holshouser, 2015). In summary, there is a balance on how double-cropping could spread fixed inputs over a large volume of output (wheat + soybean yield) while taking into account increasing production costs and augmenting income by having a second crop (Farno et al., 2002; Kelley, 2003; Holshouser, 2015). The yield gap between full-season and double-cropping soybeans becomes more evident as latitudes increase, due to reduced growing season, which may as well affect seed quality (Koenning, 1991; Caviglia et al., 2011). Reduction in yield in double-cropping soybean may be linked to different factors, including shorter growing seasons, reduced water and nutrient availability, and the presence of undecomposed and undistributed wheat residue (Caviglia et al., 2011; Holshouser, 2015).

Looking to the future, climate change and increasing temperatures may allow farmers to practice double-cropping at even higher latitudes, which may alleviate some of the predicted negative impacts of climate change on soybean and corn (Seifert and Lobell, 2015). Several simulations predict an increase in areas suitable for double-cropping by 0.35 million km^2^ (Zabel et al., 2014; Seifert and Lobell, 2015). Zabel et al. (2014), using predictive models, estimated that rises in prevailing temperatures of 2, 3, and 4°C will expand areas with potential for double-cropping by 8,370 (55.1%), 11,630 (89.5%), and 13,580 (89.5%) km^2^, respectively (% value of current double-cropping total area: 15,180 km^2^). A large part of these areas with the potential for wheat–soybean acreage increase is in Illinois, Indiana, and Eastern Ohio. In the current study, winter wheat led to a reduction in SCN egg densities compared with fallow at R1 growth stage of soybean and after soybean harvest. Farmers growing full-season soybeans in South/Central Illinois and similar regions may benefit from introducing wheat as a winter crop. Incorporating wheat into double-cropping systems can help growers to maintain SCN field populations under damage threshold and reduce costs and losses caused by SCN, the main yield-limiting biotic factor in U.S. soybean production. This study is meant to be a first step toward a better understanding of the mechanisms that govern the effect of wheat on SCN populations. Future research will explore the potential effects of wheat and related plant exudates on soil microbial communities and associated impacts on SCN populations.

## Data Availability Statement

The raw data supporting the conclusions of this article will be made available by the authors, without undue reservation.

## Author Contributions

JB, TW, and DD planned, conducted, and supervised field trials. JB and TW applied pesticides and collected the soil samples. LR wrote the manuscript with editing assistance from MP, AF, and JPB. LR designed the figures and formatted and submitted the manuscript. All authors read and agreed to the published version of the manuscript.

## Conflict of Interest

TW was employed by the company Wyciskalla Consulting. JB was employed by JCB Ag Research. DD was employed by Davidson Consulting. The remaining authors declare that the research was conducted in the absence of any commercial or financial relationships that could be construed as a potential conflict of interest.
