# Supplementary material for: Impact of Wheat on Soybean Cyst Nematode Population Density in Double-Cropping Soybean Production
Source: Front Plant Sci. 2021 May 10;12:640714. doi: 10.3389/fpls.2021.640714 (PMC8141799; doi:10.3389/fpls.2021.640714)
Supplement: Supplementary file 1 [file Data_Sheet_1.docx]

Supplementary Material

# Supplementary Figures

**

**Supplemental Figure 1**: (A) A picture showing strips separating treatments in the field trial. (B) Field trial setup used for each location, showing strips and subplots.

## Supplementary Tables

**Supplemental Table 1**: HG type test results for field populations at the beginning of trials.

| **Field** | **IL County** | **SCN Levels** | **HG Type** |
| --- | --- | --- | --- |
| 1 | Perry | Moderate | 2.5.7 |
| 2 | St. Clair | Moderate | 2.5.7 |
| 3 | Washington | Low | 2.5.7 |
| 4 | Fayette | Low | 2.5.7 |
| 5 | Franklin | Low | 2.5.7 |
| 6 | Washington | High | 2.5.7 |
| 7 | Washington | High | 2.5.7 |
| 8 | Washington | High | 2.5.7 |
| 9 | Washington | Moderate | 7 |

**Supplemental Table 2**: Effect of SCN initial population density (I - low, moderate, or high^1^) and winter rotation (W - fallow or wheat) on the SCN population ratio comparing end of soybean season (October 2018) to soybean planting (June 18).

| **ANOVA Table** | | | |
| --- | --- | --- | --- |
| **Source** | **DF** | **F Ratio** | **P > F** |
| SCN Initial Pop. (I) | 2 | 19.166 | <.0001* |
| Winter Rotation (W) | 1 | 12.263 | 0.0006 |
| I*W | 2 | 1.202 | 0.3036 |
| **Mean Comparison** | | | |
| **Factor** | **Level** | **Means** | |
| Initial Pop. | Low | 9.441 A | |
|  | Moderate | 1.183 B | |
|  | High | 1.015 B | |
| Winter Rotation | Fallow | 4.999 A | |
|  | Wheat | 1.811 B | |

^1^Means followed by the same letter in the column are not different following Tukey’s HSD Test (p=0.05). ^2^SCN population densities were defined as high SCN when having > 6,500 eggs/100 cm^3^ of soil, moderate for 2,000 - 6,500 eggs/100 cm^3^ and low with <2,000 eggs/100 cm^3^.

**Supplemental Table** 3: List of all soybean and wheat varieties, soil descriptions and pesticides used during field trials.

| **Field** | **2017 Soy**  **Variety** | **Trait^1^** | **MG^2^** | **Fertilizer** | **Rate** | **Wheat**  **Variety** | **2018 DC** | **Trait^4^** | **MG^5^** | **DC Soy** | **Soil Number** | **Soil** | **Wheat** | **Rate^7^** | **DC Soy** | **Rate^7^** |
| --- | --- | --- | --- | --- | --- | --- | --- | --- | --- | --- | --- | --- | --- | --- | --- | --- |
|  |  |  |  |  | **(Kg ha^-1^)** |  | **Soy Variety** |  |  | **Planting** | **Common name** | **Description** | **Pesticides^6^** |  | **Pesticides^6^** |  |
| 1 | Pioneer  37T09L | LL | 3.7 | Fall DAP | 224.2 | Pioneer  25R77 | Pioneer  41T79L | LL | 4.1 | 6/13/18 | 3A - Hoyleton | Fine, montmorillonitic, mesic Aquollic Hapludalfs | Harmony^H^ | 35.01 | Gramoxone^H^ | 560.43 |
|  |  |  |  |  |  |  |  |  |  |  |  |  |  |  | Medal^H^ | 1923.22 |
|  |  |  |  | Fall Potash | 224.2 |  |  |  |  |  |  |  | Prosaro^F^ | 231.17 | Glory^H^ | 499.13 |
|  |  |  |  |  |  |  |  |  |  |  |  |  |  |  | Liberty^H^ | 1.45 |
|  |  |  |  | Spring Actual N | 112.1 |  |  |  |  |  |  |  | Silencer^I^ | 33.63 | Warrant^H^ | 1259.27 |
|  |  |  |  |  |  |  |  |  |  |  |  |  |  |  | Clethodim^H^ | 175.12 |
| 2 | AgVenture  43M4LL | LL | 4.3 | Fall DAP | 168.1 | AgriMaxx  473 | AgVenture  41B5LL | LL | 4.1 | 6/19/18 | 90A - Bethalto silt loam | Fine-silty, mixed, superactive, mesic Udollic Endoaqualfs | Harmony^H^ | 35.01 | Select^H^ | 225.36 |
|  |  |  |  | Fall Potash | 168.1 |  |  |  |  |  |  |  | Tilt^F^ | 112.09 | Liberty^H^ | 1.45 |
|  |  |  |  | Spring Actual N | 100.9 |  |  |  |  |  |  |  | Prosaro^F^ | 231.17 | Pummel^H^ | 1471.11 |
| 3 | Pioneer  45T74X | Xtend | 4.5 | Fall DAP | 224.2 | Pioneer  25R77 | Pioneer  48A60X | Xtend | 4.8 | 6/16/18 | 13B - Bluford silt loam | Fine, montmorillonitic, mesic Aeric Ochraqualfs | Elevore^H^ | 5.02 | FeXapan^H^ | 864.87 |
|  |  |  |  | Fall Potash | 224.2 |  |  |  |  |  |  |  |  |  | Everprex^H^ | 1017.18 |
|  |  |  |  | Spring Actual N | 112.1 |  |  |  |  |  |  |  |  |  |  |  |
| 4 | FS HiSoy  39X7 | Xtend | 3.9 | Fall DAP | 168.1 | Blend^8^ | Asgrow  43X6 | Xtend | 4.3 | 7/5/18 | 3A - Hoyleton silt loam (Reps 1 and 2) | Fine, montmorillonitic, mesic Aquollic Hapludalfs | Harmony^H^ | 35.01 | Prefix^H^ | 1852.92 |
|  |  |  |  | Fall Potash | 168.1 |  |  |  |  |  | 13A - Bluford silt loam (Rep 3) | Fine, montmorillonitic, mesic Aeric Ochraqualfs |  |  | Select Max^H^ | 101.91 |
|  |  |  |  | Spring Actual N | 100.9 |  |  |  |  |  |  |  |  |  | Glyphosate^H^ | 280.32 |
| 5 | Pioneer  35T58R | RR | 3.5 | Fall DAP | 224.2 | Pioneer  25R47 | Pioneer  50A78L | LL | 5 | 6/16/18 | 12A - Wynoose silt loam | Fine, montmorillonitic, mesic Typic Albaqualfs | Harmony^H^ | 35.01 | Gramoxone^H^ | 560.43 |
|  |  |  |  | Fall Potash | 224.2 |  |  |  |  |  |  |  | Warrior^I^ | 33.63 |  |  |
|  |  |  |  | Spring Actual N | 112.1 |  |  |  |  |  |  |  | Headline^F^ | 16.46 | Liberty^H^ | 1.45 |
|  |  |  |  |  |  |  |  |  |  |  |  |  | Kocide^F^ | 672.52 |  |  |
| 6 | Asgrow  42X6 | Xtend | 4.2 | Fall DAP | 224.2 | Beck's  113 | Asgrow  46X6 | Xtend | 4.6 | 6/20/18 | 3A - Hoyleton silt loam | Fine, montmorillonitic, mesic Aquollic Hapludalfs | Harmony^H^ | 35.01 | XtendiMax^H^ | 560.43 |
|  |  |  |  | Fall Potash | 196.1 |  |  |  |  |  |  |  | Silencer^I^ | 33.63 | Glyphosate^H^ | 280.32 |
|  |  |  |  |  |  |  |  |  |  |  |  |  | Quilt Xcel^F^ | 231.17 | Anthem Max^H^ | 0.29 |
|  |  |  |  | Spring Actual N | 112.1 |  |  |  |  |  |  |  | Prosaro^F^ | 231.17 | Warrant^H^ | 1259.27 |
|  |  |  |  |  |  |  |  |  |  |  |  |  | Sultrus^I^ | 42.60 | Sultrus^I^ | 24.66 |
| 7 | Pioneer  48T27X | Xtend | 4.8 | Fall DAP | 224.2 | Pioneer  25R61 | Pioneer  48A60X | Xtend | 4.8 | 6/21/18 | 882A - Oconee-Darmstadt-Coulterville silt loam Complex | Fine, montmorillonic, mesic Udollic Ochraqualfs | Elevore^H^ | 5.02 | FeXapan^H^ | 864.87 |
|  |  |  |  | Fall Potash | 224.2 |  |  |  |  |  |  | Fine-silty, mixed, mesic Albic Natraqualfs |  |  | Everprex^H^ | 1017.18 |
|  |  |  |  | Spring Actual N | 112.1 |  |  |  |  |  |  | Fine-silty, mixed, mesic Aeric Ochraqualfs |  |  |  |  |
| 8 | Credenz  CZ3841LL | LL | 3.8 | Fall DAP | 224.2 | FS  622 | Beck's  494L4 | LL | 4.9 | 6/19/18 | 517A - Marine silt loam | Fine, montmorillonitic, mesic Aeric Albaqualfs | Nimble^H^ | 49.05 | Liberty^H^ | 1.45 |
|  |  |  |  |  |  |  |  |  |  |  |  |  | Harmony^H^ | 35.01 |  |  |
|  |  |  |  | Fall Potash | 224.2 |  |  |  |  |  |  |  | Silencer^I^ | 33.63 |  |  |
|  |  |  |  | Spring Actual N | 112.1 |  |  |  |  |  |  |  | Prosaro^F^ | 231.17 |  |  |
|  |  |  |  |  |  |  |  |  |  |  |  |  | Palisade^GH^ | 112.09 |  |  |
| 9 | Credenz  CZ3841LL | LL | 3.8 | Fall DAP | 224.2 | AgriMaxx  463 | Beck's  494L4 | LL | 4.9 | 6/14/18 | 882A - Oconee-Darmstadt-Coulterville silt loam Complex | Fine, montmorillonic, mesic Udollic Ochraqualfs  Fine-silty, mixed, mesic Albic Natraqualfs  Fine-silty, mixed, mesic Aeric Ochraqualfs | Nimble/Harmony | 35.01 | Liberty^H^ | 1.45 |
|  |  |  |  | Fall Potash | 224.2 |  |  |  |  |  |  |  | Silencer | 33.63 |  |  |
|  |  |  |  | Spring Actual N | 112.1 |  |  |  |  |  |  |  | Prosaro^F^ | 231.17 |  |  |
|  |  |  |  |  |  |  |  |  |  |  |  |  | Palisade^GH^ | 112.09 |  |  |

^1^2017 Soybean variety traits (LL: Liberty Link; RR: Roundup Ready); ^2^2017 soybean variety maturity group (LL: Liberty Link); ^3^Fertilizer rate (1 lb A^-1^ = 1.12085 Kg ha^-1^); ^4^2018 DC soybean traits; ^5^2018 DC soybean maturity group; ^6^Pesticides include herbicides (H), fungicides (F), insecticides (I) and growth regulators (GH); ^7^Rate in total grams of active ingredient per hectare (grams of ai ha^-1^). ^8^Blend of FS wheat varieties (FS 622 and FS 616).
