# Supplementary figures and images for: Impact of Wheat on Soybean Cyst Nematode Population Density in Double-Cropping Soybean Production
Source: Front Plant Sci. 2021 May 10;12:640714. doi: 10.3389/fpls.2021.640714 (PMC8141799; doi:10.3389/fpls.2021.640714)

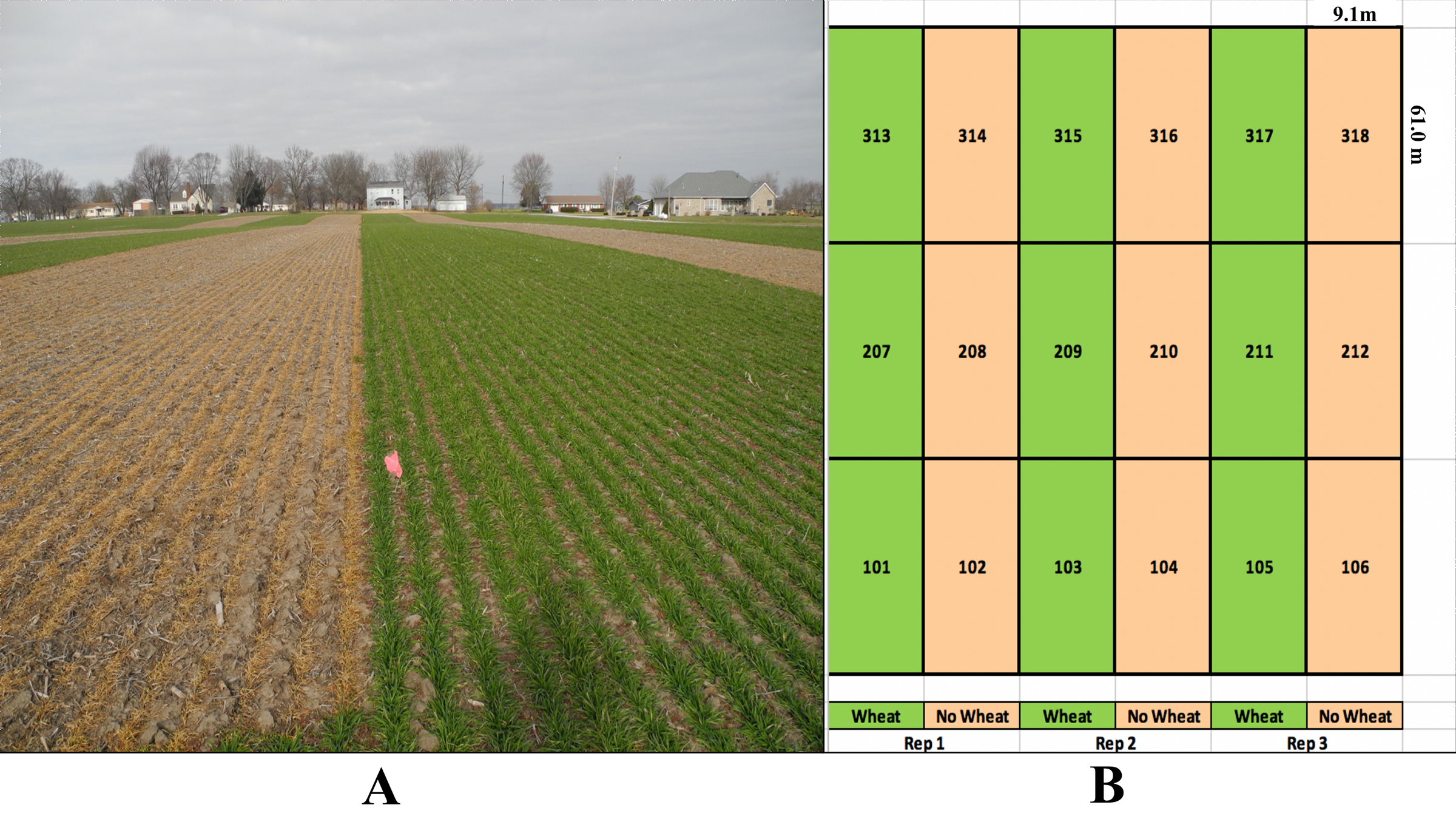

Supplement: Supplementary file 2 [file Image_1.TIFF]
